# Challenges and Opportunities While Developing a Group A Meningococcal Conjugate Vaccine Within a Product Development Partnership: A Manufacturer's Perspective From the Serum Institute of India

**DOI:** 10.1093/cid/civ500

**Published:** 2015-11-09

**Authors:** Prasad S. Kulkarni, Muriel Socquet, Suresh S. Jadhav, Subhash V. Kapre, F. Marc LaForce, Cyrus S. Poonawalla

**Affiliations:** 1 Serum Institute of India, Ltd, Pune; 2 PATH, Geneva, Switzerland

**Keywords:** Serum Institute of India Ltd, Product Development Partnership, MenAfriVac

## Abstract

***Background.*** In 2002, the Meningitis Vaccine Project (MVP) chose the Serum Institute of India, Ltd (SIIL), as its manufacturing partner to establish a product development partnership (PDP) with the Meningitis Vaccine Project (MVP). MVP was a collaboration between PATH and the World Health Organization (WHO) to develop meningococcal conjugate vaccines for sub-Saharan Africa.

***Method.*** From the outset, SIIL recognized that a partnership with MVP carried some risk but also offered important opportunities for accessing new conjugate vaccine technology and know-how. Over 3 years, SIIL successfully accepted technology transfer for the group A meningococcal polysaccharide from SynCo Bio Partners and a conjugation method from the US Food and Drug Administration.

***Results.*** SIIL successfully scaled up production of a group A meningococcal conjugate vaccine that used SIIL tetanus toxoid as the carrier protein. Phase 1 studies began in India in 2005, followed by phase 2/3 studies in Africa and India. A regulatory dossier was submitted to the Indian authorities in April 2009 and WHO in September 2009. Export license was granted in December 2009, and WHO prequalification was obtained in June 2010. Vaccine was introduced at public scale in Burkina Faso that December. The group A meningococcal conjugate vaccine was named MenAfriVac*,* and is the first internationally qualified vaccine developed outside of big pharma.

***Conclusions.*** The project proved to be a sound investment for SIIL and is a concrete example of the potential for PDPs to provide needed products for resource-poor countries.

Making and licensing a new vaccine is a complicated task, and new vaccine development has largely been the province of multinational companies often referred to as Pharma. This product development model is expensive and estimated to cost US$500–$750 million per vaccine [1]. Quite understandably, the projected profitability of the final product plays a major role in determining what vaccines are developed. However, this business model poses a major limitation for diseases that are unique to poor countries.

The problem of group A meningococcal (MenA) meningitis in sub-Saharan Africa is one such example. Meningitis due to MenA has not been an issue in developed countries since World War II. However, it has continued as an important public health challenge in sub-Saharan Africa for more than a century [2]. Despite major outbreaks every 10 years or so and annual epidemics, there were no Pharma plans to develop a MenA conjugate vaccine for Africa. Rather, in the 1990s, Pharma companies were committed to the development of group C meningococcal conjugate vaccines for the United Kingdom and multivalent meningococcal polysaccharide and conjugate vaccines for developed country markets [3]. During meningitis outbreaks, African countries relied on reactive vaccination campaigns using polysaccharide vaccines that did not protect children <2 years of age and provided no long-term protection in older age groups [4]. As a result, despite using more than a hundred million doses of A/C polysaccharide vaccine in the region from 1999 to 2003, MenA epidemics were still occurring [5].

To meet this challenge, The Meningitis Vaccine Project (MVP) was created in 2001, with the support of the Bill & Melinda Gates Foundation (BMGF), as a partnership between the World Health Organization (WHO) and PATH, a Seattle-based global health nongovernmental organization. The project had a single goal: developing, testing, licensing, and introducing an affordable MenA conjugate vaccine for Africa [6]. The Serum Institute of India, Ltd (SIIL), partnered with MVP and succeeded in manufacturing and licensing MenAfriVac in 2010 as the first internationally qualified vaccine that was developed outside of Pharma. Much has been written describing the scientific parameters of the vaccine, the clinical trials, and the vaccine's successful introduction [7–10]. This article describes the journey taken by SIIL and offers a manufacturer's insight into the risks, challenges, and opportunities associated with the development of this new vaccine within a product development partnership (PDP).

## ORIGIN OF PRODUCT DEVELOPMENT PARTNERSHIPS AND THE MVP

To address the need for vaccines in developing countries, PDPs have emerged as important entities [11]. PDPs attract private sector companies to the area of neglected diseases and have been described as follows: “The PDPs have in common a strategy of promoting collaboration between public and private sector institutions in developed and developing countries, using each partner's strengths toward a common goal. The PDPs are nonprofit entities operating with philanthropic funds and form collaborative partnership with private sector health technology corporations to design and implement product development programs for specific health technologies” [12]. The goal of PDPs is to accelerate the development of safe, effective, and affordable drugs or vaccines for the people who need them the most.

After the dreadful 1996–1997 MenA meningitis epidemic, with >180 000 reported cases, African Ministers of Health approached WHO for help. WHO convened a series of international meetings that concluded that there was a pressing need for a MenA conjugate vaccine for Africa. In June 2001, WHO and PATH received a grant from BMGF to create a PDP, called MVP, that would develop a MenA conjugate vaccine [6]. MVP was one of the first BMGF-funded PDPs created to develop a vaccine. After detailed consultations with African public health officials at the time, there was agreement that the price of the vaccine had to be about US$0.50 per dose to ensure that the vaccine's use after introduction would be sustainable.

Initially MVP envisioned that a Pharma company would partner with MVP to manufacture the vaccine, as several manufacturers were working on multivalent meningococcal conjugate vaccines containing group A conjugates. However, the proposed vaccine price proved to be a barrier to working with Pharma. Instead, in the spring of 2002, MVP decided to partner with a developing country vaccine manufacturer. After careful evaluation of companies that manufactured at least one WHO prequalified product, MVP's expert panel chose SIIL as its manufacturing partner.

## SERUM INSTITUTE OF INDIA, LTD

SIIL is a private Indian vaccine manufacturing company based in Pune, India, that is committed to manufacture and supply of affordable vaccines to Indian and international public health agencies. The company was established in 1967 when it started making antitetanus serum. At the time, tetanus was rampant in India and only expensive imported antisera in limited quantities were available, which resulted in many deaths. In 1971, SIIL released its first vaccine, tetanus toxoid. This was followed by diphtheria, tetanus, and pertussis (DTP) vaccines; measles, mumps, and rubella (MMR) vaccines; and BCG, hepatitis B, *Haemophilus influenzae* type b (Hib), rabies, and influenza vaccines [13]. Twenty of its vaccines are now prequalified by WHO and supplied to international agencies such as the United Nations Children's Fund (UNICEF) and the Pan American Health Organization (PAHO). SIIL had a long tradition of supplying vaccines to resource-limited countries through UNICEF; hence, MVP's goal of broad use of the new MenA conjugate vaccine at an affordable price was not a problem, because it fitted into SIIL's business model of supplying quality vaccines at low cost and in high quantities. SIIL is the largest global supplier of DTP/hepatitis B/Hib and MMR vaccines.

## RISKS AND CHALLENGES

### The SIIL/MVP PDP as a New Development Model

Under normal circumstances, SIIL, as a private company, would have chosen their partners. Previously, SIIL had not worked with multiple partners; under the new PDP model, SIIL did not control partner selection. To no one's surprise, there were occasional difficulties in trying to manage communications among multiple partners that included the Center for Biologics Evaluation and Research (CBER), US Food and Drug Administration (FDA), SynCo Bio Partners, Aerial Laboratories, Illkirch-Graffenstaden, National Institute of Biological Standards and Control (NIBSC), US Centers for Disease Control and Prevention (CDC), UK Health Protection Agency (now known as Public Health England), University of Cape Town, South Africa, and several MVP consultants.

The collaboration between many public/private partners was a critical task as the activities were interdependent. For example, if delivery of MenA polysaccharide from SynCo Bio Partners was delayed, the development work either stopped or had to be reprogrammed. SIIL remained flexible while facing the usual ups and downs associated with vaccine development. For example, a partner was initially selected to develop and to transfer a conjugation technology to SIIL, but it withdrew from the project just before the technology transfer was to begin. This event caused great consternation that was quickly resolved when new intellectual property for a conjugation method at the FDA was identified and licensed to PATH, which then sublicensed the technology to SIIL. Nevertheless, the change in the sourcing for the conjugation method delayed the project by a full year.

### Skepticism and Criticism of the Choice of SIIL as the Manufacturing Partner

In the early phase of the PDP, SIIL and MVP faced skepticism and criticism from Pharma and some members of international organizations about the selection of a developing country manufacturer rather than a Pharma company. Despite SIIL's success in getting vaccines prequalified by WHO and supplying vaccines to many countries through UNICEF and PAHO, some critics voiced the view that the SIIL choice would result in either outright failure or a less than ideal vaccine. MVP and SIIL were well aware of these apprehensions and, in particular, the concerns surrounding the licensure of the product in India. However, SIIL and MVP felt that strict adherence to the European Medicines Agency (EMA), the Drug Controller General of India (DCGI), and WHO requirements would ensure that the vaccine would meet the highest international standards and that rigorous adherence to these standards would calm fears about the quality of the product being developed.

### Complex Commercialization Agreements and Contracts

Because PDPs are independent legal entities, they must adhere to the laws and regulations of the country where they are based; hence, French, US, and Indian laws and regulations had to be followed when appropriate [14]. Commercialization agreements were negotiated by PATH's legal department on behalf of MVP, and negotiating these contracts was complex and time consuming. For example, the commercial agreement between SIIL and PATH was complicated because the agreement guaranteed priority for initial supplies of the vaccine to meningitis belt countries and outlined cost parameters for public sector sales at less than US$0.50 per dose. Closing the agreement was a major legal and administrative challenge, and the process took 2 years before it was finalized. Nonetheless, development work proceeded as though a formal agreement was in place.

### Technical Expertise at SIIL and Intellectual Property Issues

Before 2002, SIIL had little experience in manufacturing conjugate vaccines, although it had begun a technology transfer for a Hib conjugate vaccine from the Rijksinstituut voor Volksgezondheid en Milieu in Bilthoven, the Netherlands. Despite the initial paucity of technical resources, SIIL accepted the challenge because the goal of the project was aligned with SIIL's desire to develop expertise in polysaccharide conjugate vaccines and was also consonant with SIIL's commitment to social responsibility.

PATH negotiated a contract with SynCo Bio Partners for the production of vaccine grade group A polysaccharide. The agreement stipulated that technology transfer of the entire method was an option available to MVP. The option was exercised in 2005 when a formal technology transfer of the production and purification of the MenA polysaccharide was transferred from SynCo to SIIL. Similarly, a partnership was established between PATH/CBER and the FDA for a conjugation technology that had been developed by Drs Robert Lee and Carl Frasch [15]. The new conjugate vaccine technology was owned by the FDA and was protected by patents that were filed worldwide, except in Africa. PATH negotiated a nonexclusive license from the US National Institute of Health's Office of Technology Transfer for the Lee/Frasch conjugation method [14]. PATH then sublicensed the technology to SIIL for development of the MenA conjugate vaccine. The technology transfer of the Lee/Frasch conjugation method began in December 2003 when a team of SIIL scientists visited the CBER laboratories. The CBER conjugation technology was at laboratory scale and was transferred to SIIL for further development and industrial scale-up. A second key raw material was the carrier protein tetanus toxoid, which was already being produced at industrial scale at SIIL.

Because all vaccine lots produced in India must be released by the National Control Laboratory (ie, Central Drugs Laboratory [CDL], Kasauli, India), its representatives along with SIIL quality control personnel traveled to Potters Bar, United Kingdom, for training at NIBSC. Several experimental lots were then manufactured and tested at NIBSC and Kasauli, and the first Good Manufacturing Practice lot of MenA conjugate vaccine was produced in 2004.

Throughout the vaccine development, a pool of expert consultants with broad expertise that spanned all aspects of conjugate vaccine production and formulation was available to SIIL. Consultants regularly interacted with SIIL technical staff and provided know-how that included research and development, manufacturing, scale-up, production capacity, quality systems, clinical studies, and regulatory activities.

### Clinical Development

Early in the vaccine development process, it was agreed that the clinical development of the vaccine would adhere to EMA and WHO norms. The clinical program was conducted in India and in sub-Saharan African countries. Study protocols were developed collaboratively with MVP and included a review by WHO and the MVP Project Advisory Group that consisted of senior African public health authorities. Multiple face-to-face meetings, teleconferences, and email exchanges facilitated the development and consensus with study documents. Clinical research organizations in India and Africa were identified and helped manage operations and data management. MVP shouldered the cost of all vaccine trials (about US$37 million).

In 2004, animal toxicology studies were done in an Indian Good Laboratory Practice laboratory. Because the vaccine was intended for women of childbearing age, special fertility and reproductive toxicity studies were done. These trials assessed low, medium, and high doses of the vaccine in accordance with the EMA guidelines and those listed in the Indian Schedule Y of Drugs and Cosmetics Act. All results pointed to a very low potential of toxicity. Last, meetings were held with the national regulatory authority, DCGI, to review the entire project and the timelines. The overall clinical plan that included a phase 1 study in India was approved by the DCGI, with the suggestion that a phase 2 study in 2- to 10-year-olds also be done in India.

Results of the clinical trials have been published and showed that the new MenA conjugate vaccine was safe, highly immunogenic, and superior to licensed polysaccharide vaccines [8, 16–18].

### Regulatory Process

From January 2008 to April 2009, SIIL and MVP worked to prepare a regulatory dossier that was submitted in April 2009 to the DCGI. At that time, the DCGI was under review by WHO, and Health Canada assisted DCGI in assessing the MenA conjugate vaccine dossier. In addition, WHO headquarters (HQ) was contacted and agreed to review the MenA conjugate vaccine dossier in parallel given the urgency of the public health need (2008 and 2009 saw major MenA epidemic years in Nigeria, with a final tally of >70 000 cases). A joint inspection of SIIL MenA conjugate vaccine production facilities by DCGI and Health Canada was conducted in 2009.

One major finding of the inspection was the change in the site of production for the MenA polysaccharide. Clinical data in the dossier were based on a MenA conjugate vaccine that used group A polysaccharide made at SynCo Bio Partners in Amsterdam. The technology for making group A polysaccharide had been transferred to SIIL, and SIIL had succeeded in making high-quality group A polysaccharide that was shown by nuclear magnetic resonance testing to be indistinguishable from the group A polysaccharide made at SynCo Bio Partners. The DCGI agreed that the physicochemical characteristics of the A polysaccharide made at SIIL in Pune were the same as those of the vaccine made at SynCo Bio Partners. Nonetheless, the MenA conjugate vaccine using the group A polysaccharide made at SIIL was not accepted by DCGI.

The problem was discussed in detail between DCGI, SIIL, PATH, and WHO. After intense deliberations, it was decided that the new MenA conjugate vaccine that used group A polysaccharide made at SIIL should undergo animal toxicity studies and a clinical trial. Animal toxicity studies were conducted and no toxicity was demonstrated. Once these data had been submitted to the DCGI, the SIIL vaccine was licensed on 24 December 2009 solely for use in Africa pending the results of an Indian clinical study in 2- to 10-year-olds. In January 2010, the vaccine, PsA-TT, was given the brand name MenAfriVac. WHO continued its review of the dossier, and WHO prequalification for use in 1- to 29-year-olds was granted in June 2010 [7]. Subsequently, after completion of a phase 3 trial in India, licensure for Indian use was also granted in October 2011.

## OPPORTUNITIES

### Access to Conjugation Intellectual Property and Conjugate Vaccine Know-how

As previously mentioned, the MVP/SIIL partnership facilitated the transfer of a new conjugate vaccine technology. Access to this intellectual property was a unique opportunity for SIIL and it was capitalized upon. SIIL scientists absorbed the FDA technology and, over time, improved the conjugation yields and scaled up the manufacturing process. Not unexpectedly, manufacturing problems surfaced and were resolved collaboratively during regularly scheduled conferences and site visits with MVP consultants. More recently, the enhanced SIIL expertise in carbohydrate conjugation that followed the PsA-TT development has helped expand SIIL's product pipeline, which now includes multivalent pneumococcal and meningococcal vaccines.

### Parallel Activities Mounted by MVP That Were of Use to SIIL

A second major advantage to SIIL from the MVP PDP was the continuation of a series of important parallel activities by MVP such as supporting the WHO/MVP Inter-country Support Team in Ouagadougou, Burkina Faso, investing in enhanced meningitis surveillance, and commissioning studies whose results would markedly ease the introduction of product when it was available. For example, as early as 2006 and while the phase 1 study results were being finalized, the planning for vaccine introduction of the MenA conjugate vaccine commenced. With most vaccines, this work is not usually begun until phase 2/3 results indicate that the product will be licensed. SIIL also benefited from the high visibility that the project enjoyed at WHO HQ, WHO AFRO, and meningitis belt countries that rapidly linked the “new meningitis vaccine” to SIIL.

### Lowering Financial Risk at SIIL

SIIL received a total of US$6.25 million to support its development work. These funds paid for salaries, equipment, disposables, and related administrative and travel costs. In addition, MVP assumed many of the costs that traditionally would be borne by a manufacturer developing a new product. For example, MVP supported the clinical trial costs in Africa and India (about US$37 million) and paid for the regulatory costs incurred in the development and the filing of the PsA-TT regulatory dossiers (about US$3 million). Nonetheless, SIIL made substantial investments over and above funds supplied by MVP and invested US$7.4 million of its own funds to improve manufacturing facilities that were dedicated to the group A conjugate vaccine.

### Close Collaboration With WHO HQ, WHO AFRO, UNICEF, and Gavi

As mentioned previously, the decision to develop a MenA conjugate vaccine arose from a specific policy action by WHO following international meetings organized between 1999 and 2001. Vaccine development for the MenA conjugate vaccine at SIIL adhered to WHO technical requirements for conjugate vaccines. All clinical protocols and the regulatory dossier were reviewed by WHO. Last, the introduction of MenAfriVac took place within a complex array of WHO HQ, WHO AFRO, Gavi, and individual country priorities that was simplified into a prioritization exercise led by WHO [19].

At a transfer price of US$0.50 per dose, SIIL did not anticipate a robust financial return on its investment in a new production facility, person-time, and opportunity cost. Rather, SIIL viewed its partnership with MVP as a development opportunity rather than a financial bonanza. SIIL anticipated that the acquisition of new technology and skills in developing carbohydrate conjugate vaccines would enhance the skills of their scientific team, and that this new competence could be used for other conjugate vaccines. In addition, the partnership with PATH and WHO and other partners would likely lead to other business opportunities. SIIL also understood that if the project was successful, there would be global visibility and prestige that would accrue to SIIL because of its involvement in solving an important public health problem. Lastly, the rollout work would provide important contact time with WHO HQ, WHO AFRO, African ministries of health and finance, UNICEF, Gavi, and BMGF.

The original PATH/SIIL contract stipulated that SIIL would furnish 25 million doses of the new vaccine annually. After the first year of introduction, the demand for PsA-TT immediately exceeded 50 million doses annually. WHO proved to be the honest broker, and intercountry introduction plans that had prompted vigorous discussion were negotiated without difficulty. In a parallel fashion, WHO worked closely with SIIL to ensure that country commitments and vaccine supply were harmonized.

### International Recognition

MenAfriVac has been hugely successful [9, 10], group A meningitis has virtually disappeared wherever the vaccine has been introduced. The success of MenAfriVac has brought recognition and acclaim to SIIL [20]. Over and above the commercial and business interests, the project's success has given great satisfaction to SIIL for its contribution in helping to vanquish an important public health scourge in resource-poor countries.

## CONCLUSIONS

PDPs, a new trend in public-private partnerships, have been shown to be a potential solution to help close the vaccine gap between developed and developing countries. The success of SIIL's MenAfriVac vaccine in eliminating MenA epidemics in sub-Saharan Africa is one such example. Key to the success of the MVP/SIIL partnership was transparency and an intense and close collaboration that profited from the value added from the participating partners. As the project matured, concern for the success of all partners pervaded the project and helped motivate the entire group. This openness facilitated the transfer of new technology and know-how to SIIL. SIIL technical and clinical competencies were improved as a result of the collaboration and are being used as SIIL develops new vaccines. In short, the development of MenAfriVac has proven to be a major step forward for the company.
